# A Bipartite Network Module-Based Project to Predict Pathogen–Host Association

**DOI:** 10.3389/fgene.2019.01357

**Published:** 2020-01-24

**Authors:** Jie Li, Shiming Wang, Zhuo Chen, Yadong Wang

**Affiliations:** School of Computer Science and Technology, Harbin Institute of Technology, Harbin, China

**Keywords:** BNMP, bipartite network project, pathogen, host, pathogen–host association

## Abstract

Pathogen–host interactions play an important role in understanding the mechanism by which a pathogen can infect its host. Some approaches for predicting pathogen–host association have been developed, but prediction accuracy is still low. In this paper, we propose a bipartite network module-based approach to improve prediction accuracy. First, a bipartite network with pathogens and hosts is constructed. Next, pathogens and hosts are divided into different modules respectively. Then, modular information on the pathogens and hosts is added into a bipartite network projection model and the association scores between pathogens and hosts are calculated. Finally, leave-one-out cross-validation is used to estimate the performance of the proposed method. Experimental results show that the proposed method performs better in predicting pathogen–host association than other methods, and some potential pathogen–host associations with higher prediction scores are also confirmed by the results of biological experiments in the publically available literature.

## Introduction

Pathogen–host interactions (PHIs) play a crucial role in understanding the mechanisms of infections and identifying potential targets for infection therapeutics. Therefore, various biological experimental or computing methods have been developed to test and predict the interactions between pathogens and hosts. However, it is not only time-consuming and laborious to test PHIs through biological experimentation but also costs a lot of money. Computing methods such as biological reasoning and machine learning are considered as another important approach for predicting PHIs. Three main approaches can be used to predict PHIs: biological reasoning homology-based, structure-based, and domain/motif interaction-based (Nourani et al., 2015). The basis of homology-based prediction is that the interaction between conserved homologous organisms would also be conserved. Lee et al. inferred more than 3000 H. sapiens–P. falciparum protein–protein interactions (PPIs) based on orthologous pairs, revealing that Plasmodium falciparum can utilize calcium regulatory proteins in host cells to maintain Ca2+ levels (Lee et al., 2008). Wuchty et al. used the random forest method to evaluate and filter homology-based prediction results, which further improved prediction accuracy (Stefan, 2011). Structure-based prediction assumes that a pair of proteins with similar protein structures that are known to interact may interact in the same manner. Davis et al. proposed an algorithm for predicting possible interactions based on the physical structure of the protein by scanning the genome of the pathogen and host to find structurally similar proteins (Davis et al., 2010). Aloy and Russell also proposed a method for inferring the molecular details of interactions that might occur by evaluating a pair of potentially interacting proteins on a complex of known 3D structures (Patrick and Russell, 2002). Doolittle et al. used this method to predict the interaction between HIV and human proteins, providing assistance for further trials and therapeutic intervention targets (Doolittle and Gomez, 2010). Domain/motif interaction-based prediction combines the known intraspecific PPI with the protein domain spectrum to predict the PPI between host and pathogen proteins (Dyer et al., 2007). Evans et al. used the method to predict the interaction between HIV-1 and human proteins, confirming that the linear binding motif shared by the virus and the host protein was an important part of the crosstalk between the virus and the host (Evans et al., 2009). Machine learning methods are widely used in the prediction of pathogen–host interaction relationships. Ahmed et al. used a comparison of a neural network model versus SVM for the prediction of host-pathogen PPI based on a combination of features including amino acid quadruplets, pairwise sequence similarity, and human interactome properties; they found that the neural network achieved a significant improvement in overall performance compared to a predictor using the triplets feature and that it achieved good accuracy in predicting B.anthracis–human interaction (Ahmed et al., 2018). Mei et al. proposed the AdaBoost approach to predict proteome-wide interactions between Salmonella and human proteins based on multi-instance transfer learning (Mei and Zhu, 2014). Subsequently, a new negative data sampling method based on single-class SVM was proposed to predict the protein interaction between HTLV retrovirus and Homo sapiens. Use of this method provided valuable cues for the pathogenesis of HTLV retrovirus (Mei and Zhu, 2015).

Predicting unknown relations between pathogens and hosts in advance is of great significance for detecting changes in their relations and preventing the spread of infectious diseases in hosts. The above methods are used to predict protein–protein interactions of pathogens and hosts based on protein-related information. However, in cases where protein information or other molecular information is unavailable and we only know the relations between pathogens and hosts, we need to develop a new method to predict the potential relations between pathogens and hosts based only on the relations of pathogens and hosts. Zhang et al. developed a bipartite network project (BNP) (Zhou et al., 2007) to predict the relations between an X set and Y set (two sets included in the bipartite network). The experimental results on personal recommendation shown that BNP performed much better than the most commonly used global ranking method. Chen et al. proposed a novel computational model of Bipartite Network Projection for MiRNA–Disease Association prediction (BNPMDA) (Chen et al., 2018) based on the known miRNA–disease associations, integrated miRNA similarity, and integrated disease similarity. BNPMDA could effectively predict the potential miRNA–disease associations with a high accuracy level. Sun et al. developed the NTSMDA method to predict miRNA–disease associations by integrating network topological similarity (Sun et al., 2016). NTSMDA demonstrates excellent predictive performance. Tad et al. developed an algorithm to predict missing links based on conditional probability estimation and associated, node-level features (Dallas et al., 2017). They validated this algorithm on simulated data and then applied it to a desert small mammal host-parasite network. The approach achieved high accuracy on simulated and observed data, providing a simple method for accurately predicting missing links in networks without relying on prior knowledge about the network structure. These methods are based on bipartite network models and are widely used in different fields. However, these methods not only ignore the relations of elements in the X set but also the relations of elements in the Y set, though these relations are important to predict the relations of the X set and Y set. Zhang et al. proposed a weight-based model (Zhang et al., 2015) in a dual-layer network, using the cell line similarity network, drug similarity network, and drug-cell line response network. WBSMDA (Chen et al., 2016a) employed the concepts of within-score and between-score to predict the association score in the association network. These methods consider the relations of elements in the X and Y sets from a global perspective, and collecting the information from a local perspective and then integrating them from the global perspective can detect the information in the network more comprehensively. Based on this idea, we proposed a bipartite network module-based project (BNMP) to predict pathogen–host associations by adding modular information into a bipartite network projection. Firstly, a pathogen–host bipartite network is constructed, and the distances of pathogens and hosts are computed respectively on the basis of the topological structure. Pathogens are then divided into several modules, as are hosts. Finally, the module information of pathogens and hosts, respectively, is applied to BNP to calculate the prediction score.

## Materials and Methods

### Data Collection and Pre-Processing

First, the pathogen–host interaction data were downloaded from PHI-base (Urban et al., 2017) (http://www.PHI-base.org/index.jsp), HPIDB (Ammari et al., 2016) (https://hpidb.igbb.msstate.edu/index.html), and IntAct (Sandra et al., 2014) (https://www.ebi.ac.uk/intact/). These three databases are commonly used molecular interaction databases that cover most of the molecular interaction data in open data sources. We downloaded all of the entire datasets of these three databases on September 8, 2019. These three databases provide downloads of previous version data, and researchers can select the related version for replication. Then, based on the taxonomy ID, we selected bacteria–host interaction data and deleted duplicate data from the data sets. The final dataset comprised data on 997 bacteria–host interactions, covering 243 hosts and 388 bacteria. The number of pathogens and hosts were *s* and *t*, respectively. We used them to generate the pathogen–host association matrix *A*. *A*[*p*_*i*_ ][*h*_*j*_ ]=1 means that there is a pathogen–host protein–protein interaction between the *i*th pathogen and the *j*th host, whereas *A*[*p*_*i*_ ][*h*_*j*_ ]=0 means there is no interaction between the *i*th pathogen and the *j*th host.

### Bipartite Network Projection

Here, for a bipartite network *G*(*P*,*H*,*E*) where P={*p*_1_,*p*_2_,…,*p*_*s*_ } and H={*h*_1_,*h*_2_,…,*h*_*t*_ } are pathogen and host sets respectively, *E*⊆*P*×*H* is the edge set between pathogens and hosts, and the association scores between a host and all pathogens can be calculated using the bipartite network projection (Zhou et al., 2007) (BNP) method. If we let a host *h*_*seed*_ be the seed vertex, the association scores between *h*_*seed*_ and all pathogens are as follows.

$$BNP\left(P,H,h_{seed}\right)=\left\{scp\left(p_{1}\right),scp\left(p_{2}\right),\ldots ,scp\left(p_{s}\right)\right\}$$

$$scp\left(p_{i}\right)=\sum_{j=1}^{t}A\left[p_{i}\right]\left[h_{j}\right]sch\left(h_{j}\right)/d\left(h_{j}\right)$$

$$sch\left(h_{j}\right)=\sum_{i=1}^{s}A\left[p_{i}\right]\left[h_{j}\right]A\left[p_{i}\right]\left[h_{seed}\right]/d\left(p_{i}\right)$$

where *d*(*h*_*j*_) and d(*p*_*i*_) are the degrees of the *j*th host and the *i*th pathogen, respectively. *scp*(*p*_*i*_) is the association score between *h*_*seed*_ and the *i*th pathogen, which requires *sch*(*h*_1_), *sch*(*h*_2_), …, *sch*(*h*_*t*_) as the input.

### Bipartite Network Module-Based Project

**Figure 1:**
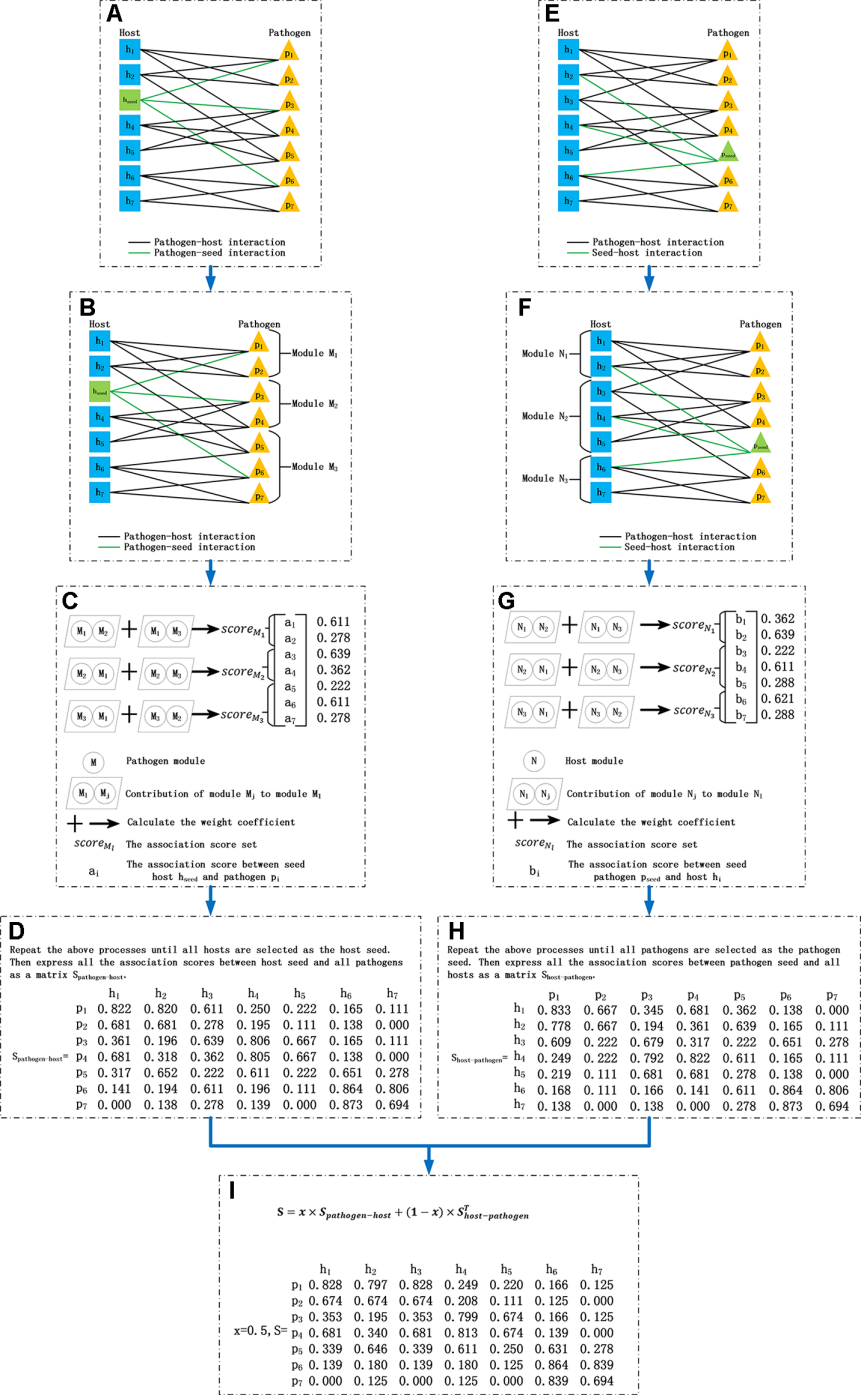
Process of the bipartite network module-based project. **(A)** Construct the pathogen–host bipartite network and choose a host as the seed vertex. **(B)** Divide the pathogen set into several modules. **(C)** Calculate the association score between the seed and pathogens in each module. **(D)** Select each host as the seed vertex in turn and repeat process **(A**–**C)** then obtain the pathogen–host association score matrix *S*_*pathogen*−*host*_ **(E)** Choose a pathogen as the seed vertex. **(F)** Divide the host set into several modules. **(G)** Calculate the association score between the seed and hosts in each module. **(H)** Select each pathogen as the seed vertex in turn and repeat process **(E**–**G)** then obtain the host–pathogen association score matrix *S*_*host*−*pathogen*_. **(I)** Integrate matrix *S*_*pathogen*−*host*_ and *S*_*host*−*pathogen*_ as the association score matrix between all pathogens and hosts.

For *G*(*P*, *H*, *E*) with *s* pathogens and *t* hosts, BNMP comprises the following steps (**Figure 1**):

1) Let a host *h*_*seed*_ be the seed vertex. Calculate the distance between two pathogens. *Dis*(*p*_*i*_,*p*_*j*_) is the distance between pathogen *p*_*i*_ and *p*_*j*_ in the following formula (**Figure 1A**), where *A*[*p*_*i*_ ] is the binary vector in the *i*th row in association matrix *A*.

$$Dis\left(p_{i},p_{j}\right)=1-\text{exp}\left(-||A\left[p_{i}\right]-A{\left[p_{j}\right]||}^{2}\right)$$

2) Divide pathogen set P={*p*_1_,*p*_2_,…,*p*_*s*_ } into *m* modules {*M*_1_,*M*_2_,…,*M*_*m*_} with *s*_1_,*s*_2_,…, and *s*_*m*_ pathogens, respectively (**Figure 1B**) where *m* is the degree of *h*_*seed*_, namely the number of pathogens associated with *h*_*seed*_, as expressed in the following formula. The intersection between two modules is empty. So $s=\sum_{l=1}^{m}s_{l}$, $M_{l}=\left\{p_{r}^{l}|p_{r}^{l}\in P,\;1\leq r\leq s_{l}\right\}.$

$$\text{m}=\sum_{i=1}^{s}A\left[p_{i}\right]\left[h_{seed}\right]$$

The process of generating *m* modules is as follows: (1) *m* pathogens associated with *h*_*seed*_ are divided into *m* modules respectively and marked as the core vertexes of the corresponding *m* modules; (2) *p*_*i*_ (i=1,2,…,s) is added to the module whose core vertex has the shortest distance from it; (3) In order to keep a balance of resources received by the *h*_*seed*_ from different modules, select *s*_*l*_−⌈*s*/*m*⌉ (⌈*s*/*m*⌉ means the rounded-up value of the result of *s*/*m*) pathogens with the furthest distance from the core vertex of *M*_*l*_ if *s*_*l*_ is larger than ⌈*s*/*m*⌉ and reassign them to other modules in which the number of pathogens is less than ⌈*s*/*m*⌉. (4) Repeat (3) until the number of pathogens in each module does not exceed ⌈*s*/*m*⌉.

3) Calculate the association score set $\bar{score_{M_{l}}}$ between *h*_*seed*_ and *M*_*l*_(*l*=1, 2,…,*m*) (**Figure 1C**).

$$\bar{score_{M_{l}}}=\frac{\sum_{1\leq j\leq m,j\neq l}w\left(M_{l},M_{j}\right)\times B_{M_{l}}}{\sum_{1\leq j\leq m,j\neq l}w\left(M_{l},M_{j}\right)}$$

where

$$w\left(M_{l},M_{j}\right)=\exp\left(-\frac{\sum_{p_{u\in M_{l}}}\sum_{p_{v\in M_{j}}}Dis\left(p_{u},p_{v}\right)}{\left|M_{l}\right|\times \left|M_{j}\right|}\right)$$

$$B_{M_{l},M_{j}}=BNP\left(\bar{M_{lj}}\;,\bar{H_{lj}}\;,\;h_{seed}\right)$$

$$B_{M_{l},M_{j}}=B_{M_{l}}\cup^{}B_{M_{j}}$$

$$\bar{M_{lj}}=M_{l}\cup^{}M_{j}$$

$$\bar{H_{lj}}=\{h_{n}|A\left[p_{k}\right]\left[h_{n}\right]=1,p_{k}\in \bar{M_{lj}}\;,1\leq n\leq t\;\}$$

*w*(*M*_*l*_,*M*_*j*_) is the weight coefficient of resources that *M*_*l*_ receive from *M*_*j*_ (*j*≠*l*). $B_{M_{l},M_{j}}$ is the association score set obtained by running the BNP algorithm on $\bar{M_{lj}}\;,\bar{H_{lj}},\;\;\text{and}\;h_{seed}$, which includes two sets: $B_{M_{l}}$ and $B_{M_{j}}$. $B_{M_{l}}$ and $B_{M_{j}}$ are the association score sets of pathogens in $B_{M_{l}}$ and $B_{M_{j}}$, respectively.

Finally, the association score set $\left\{\bar{score_{M_{1}}},\bar{score_{M_{2}}},\ldots ,\bar{score_{M_{m}}}\right\}$ between *h*_*seed*_ and all pathogens is obtained.

4) Select each host as the seed vertex in turn, and repeat the process above. Obtain *r* association score sets, and combine them to form a pathogen and host association score matrix *S*_*pathogen*−*host*_ (**Figure 1D**). Each element of *S*_*pathogen*−*host*_ is an association score of a pathogen and a host. Similarly, chose a pathogen as the seed vertex in turn, and obtain another association score matrix, *S*_*host*−*pathogen*_ (**Figures 1E–H**).

5) Finally, take the integrated value of the two matrices, *S*_*pathogen*−*host*_ and $S_{host-pathogen}^{T}$, as the association score matrix between pathogens and hosts, where *x* is a parameter to balance *S*_*pathogen*−*host*_ and $S_{host-pathogen}^{T}$ (**Figure 1I**):

$$\text{S}=x\times S_{pathogen-host}+\left(1-x\right)\times S_{host-pathogen}^{T}$$

## Results

### Performance Evaluation

Leave-one-out cross-validation (Kohavi, 1995) (LOOCV) is used to evaluate the performance of BNMP relative to previous evaluation methods (Geeleher et al., 2014; Zhang et al., 2015; Chen et al., 2016b; Sun et al., 2016 Fei et al., 2018; Le and Pham, 2018). Specifically, each known pathogen–host interaction is chosen as a test data set in turn, the remaining known interactions are chosen as the training set, and the pathogen–host association score in the training set is calculated using BNMP. After the LOOCV test process is completed, we plot the receiver operating characteristic (ROC) curve and precision recall (PR) curve and use the area under the ROC curve (AUROC) and the area under the PR curve (AUPR) to evaluate the performance of BNMP.

### Performance Analysis of BNMP

**Figure 2:**
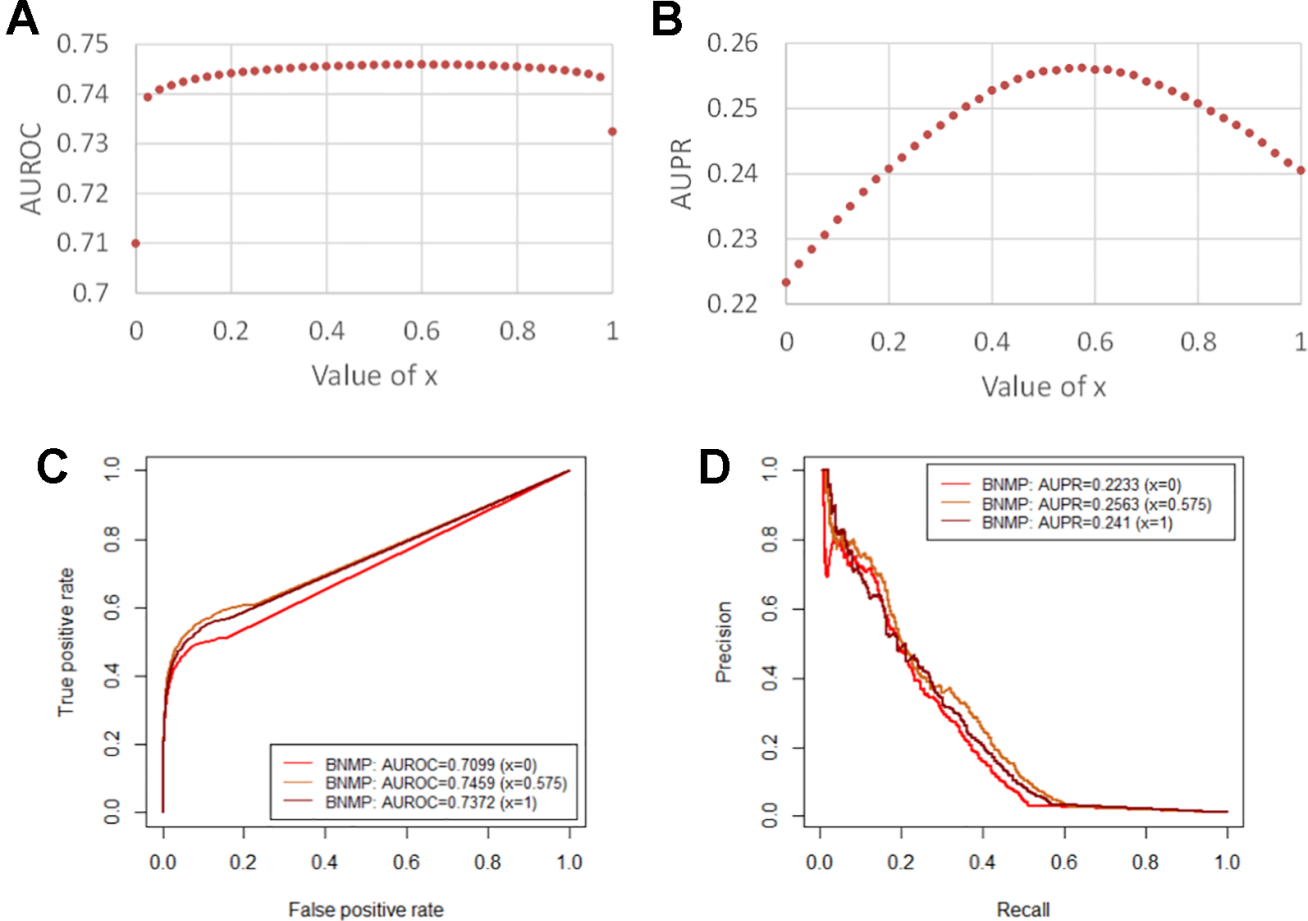
Prediction performance of BNMP with network 1. **(A)** Influence on AUROC values by different balance parameter values. **(B)** Influence on AUPR values by different balance parameter values. **(C)** ROC curves of BNMP with the different balance parameter values. **(D)** PR curves of BNMP with the different balance parameter values.

**Figure 3:**
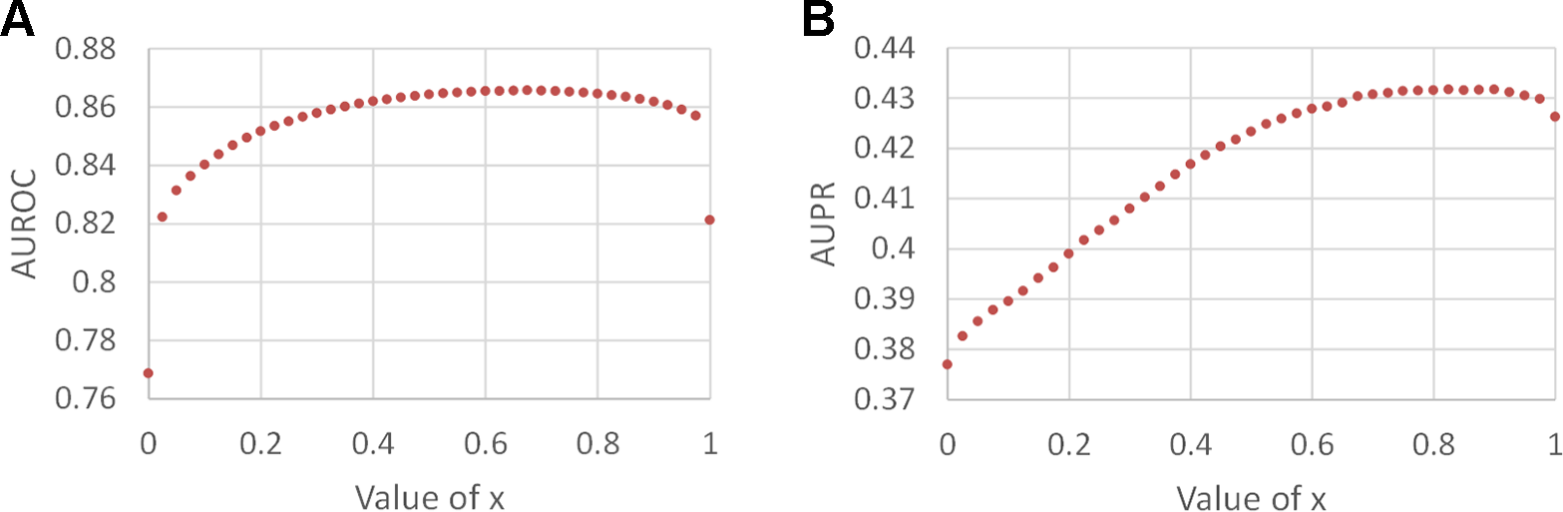
Prediction performance of BNMP with network 2. **(A)** Influence on AUROC values by different balance parameter values. **(B)** Influence on AUPR values by different balance parameter values.

We constructed the pathogen–host association network, namely network 1, which consists of 388 pathogens, 243 hosts, and 997 associations, as shown in **Table 1**. To clarify the influence of the balance parameter *x*, AUROC and AUPR values were calculated with different values of *x*, as shown in **Figures 2A** and **B**. It can be found that the prediction performance with *x*, ∈ (0, 1) is better than with *x* = 0 or *x* = 1, demonstrating the effectiveness of the integrated association score matrix. When *x* = 0.575, BNMP acquires the highest AUROC and AUPR values. We plotted the ROC and PR curves when *x* = 0,0.575, and 1, as shown in **Figures 2C** and **D**. It is noteworthy that the ROC curves take the form of an oblique upward-sloping straight line. We analyzed the results and found that more than half of the hosts are related to only one pathogen. As a result, the association scores between these hosts and pathogens are predicted to be zero in the LOOCV experiment, which has little worth for our prediction and results in the oblique upward-sloping straight line rather than a smooth ROC curve. To evaluate the prediction accuracy of BNMP on hosts (pathogens) that have more than one association with pathogens (hosts), the rows or columns with only one “1” are removed from the pathogen–host association matrix. After processing, 167 pathogens, 96 hosts, and 653 associations remained, namely network 2, and this was used to evaluate the performance of BNMP, as shown in **Table 1**. The analysis regarding *x* is shown in **Figures 3A** and **B**. When *x* = 0.675, BNMP achieves the highest AUROC value of 0.8656. When *x* = 0.825, BNMP achieves the highest AUPR value of 0.4318.

**Table 1:** The constructed network 1 and network 2.

Network	Number of pathogens	Number of hosts	Number of associations
Network 1	388	243	997
Network 2	167	96	653

### Comparison With Existing Methods

**Figure 4:**
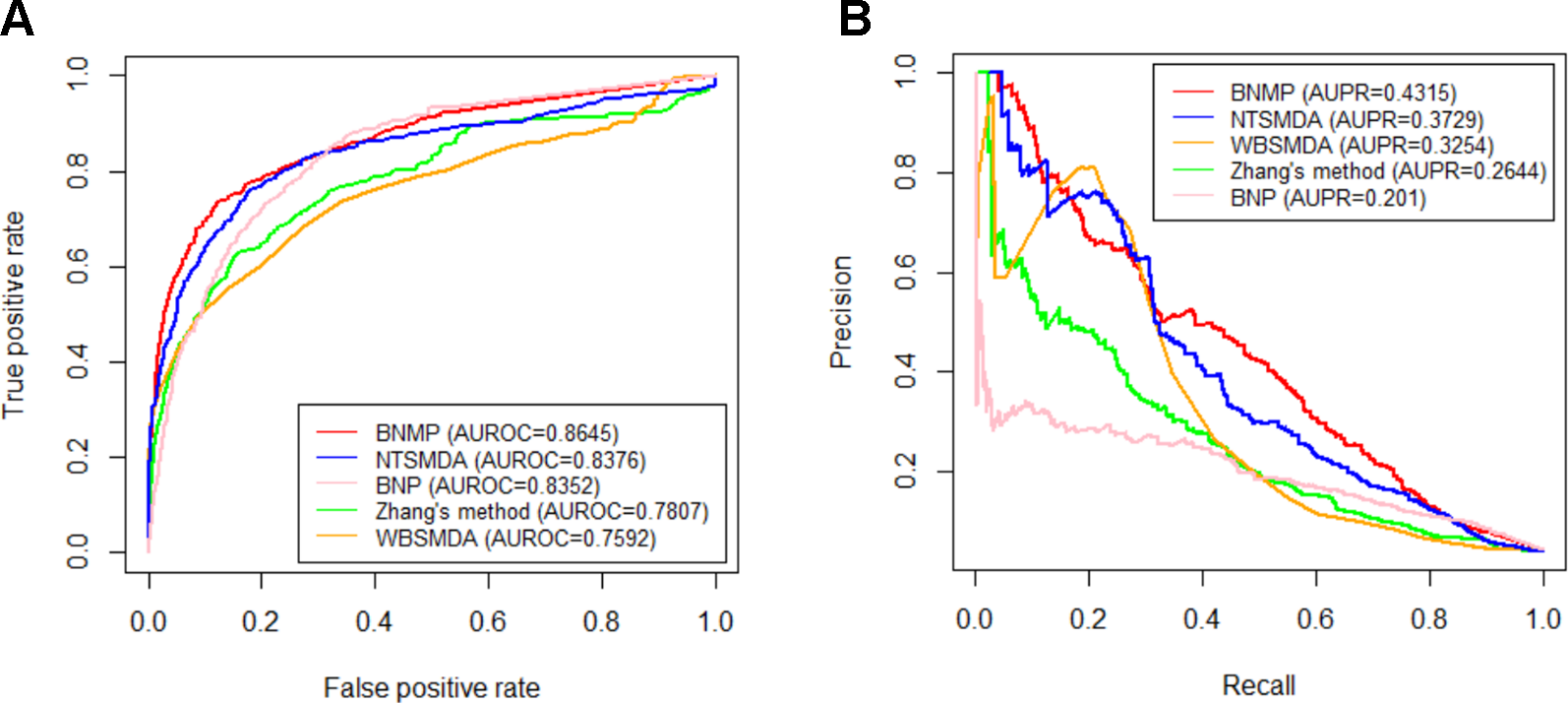
Comparison of five methods. **(A)** ROC curves. **(B)** PR curves.

**Figure 5:**
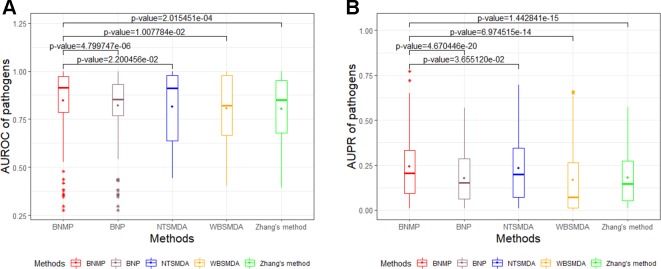
Paired *t*-test for the AUROC and AUPR values of pathogens between BNMP and other methods. **(A)** Box-and-whisker plot of AUROC values with *p*-values. **(B)** Box-and-whisker plot of AUPR values with *p*-values.

In order to further prove the effectiveness of the proposed method, BNMP is compared with four other methods : Zhang's method (Zhang et al., 2015), NTSMDA (Sun et al., 2016), WBSMDA (Chen et al., 2016a), and BNP (Zhou et al., 2007). BNMP has different prediction performance when x is different (see **Figure 3**). To ensure the fairness of the comparison, we did not select the best prediction performance of BNMP for comparison with the other four methods. Instead, we ranked the AUROC values in **Figure 3A** in descending order and selected the upper quartile (the corresponding *x* value is 0.8) for comparison with other methods. LOOCV experiments were performed with BNMP, Zhang's method, NTSMDA, WBSMDA, and BNP, and the resulting ROC and PR curves are shown in **Figure 4**. BNMP acquires an AUROC value of 0.8645, exceeding those of NTSMDA (0.8376), BNP (0.8352), Zhang's method (0.7807), and WBSMDA (0.7592). Meanwhile, BNMP obtains an AUPR value of 0.4315, exceeding those of NTSMDA (0.3729), WBSMDA (0.3254), Zhang's method (0.2644), and BNP (0.201). We also calculated the AUROC and AUPR values for each pathogen by these methods, and performed a paired *t*-test (Demišar and Schuurmans, 2006) between BNMP and the other methods (see **Figure 5**). The result is that all the *p*-values are less than 0.05, indicating that the proposed approach is a significant advance over the previous approaches and has better prediction ability.

### Validation *via* Biological Evidence

Most data sources use text mining algorithms to obtain the original interaction data. Due to the limitation of the development of pathogen–host interaction text mining algorithms, the existing open data sources can only cover a part of pathogen–host interaction data. To further test the ability of BNMP to predict potential pathogen–host associations, we rank pathogen–host pairs without relations in existing data sets according to association scores and search the public literature to see whether there is evidence that pathogens and hosts with higher association scores have relations. It is found that among the top 20 pathogen–host pairs without relations in the existing data set, biological experiments have verified that 16 pairs have associations (**Table 2**); these 16 pairs are ranked lower by the other four methods. The pair of pathogen Serratia marcescens and host Mus musculus ranks 1st. Iwaya A et al. studied the clinical application and evaluation of rapid and quantitative detection of blood Serratia marcescens by a real-time PCR assay in a mouse infection model (Iwaya et al., 2005). The pair of pathogen Cronobacter turicensis and host Mus musculus ranks 3rd. Tóthová Ľ et al. used Cronobacter turicensis to infect female mice to prove the effects of isolated Cronobacter-specific phages on renal colonization in a model of urinary tract infection in mice (Tóthová et al., 2011). The pair of pathogen Escherichia coli O157:H7 and host Mus musculus ranks 4th. Tanji Y et al. found that repeated oral administration of SP15-21-22 can effectively treat mice infected with Escherichia coli O157:H7 (Tanji et al., 2005). The pair of pathogen Acinetobacter nosocomialis and host Homo sapiens ranks 5th. Visca P et al. discussed the infection mechanism and threats of Acinetobacter nosocomialis and other Acinetobacter species to humans (Visca et al., 2011). The pair of pathogen Stenotrophomonas maltophilia and host Mus musculus ranks 6th. Bacterial adhesion to mouse tracheal mucus as the role of flagella in the adhesion process were investigated using clinical isolates of Stenotrophomonas maltophilia (Zgair and Chhibber, 2011). The pair of pathogen Sclerotinia sclerotiorum and host Nicotiana tabacum ranks 7th. Researchers carried out a preliminary evaluation of the potential of polyamine biosynthesis inhibition a strategy for the control of plant diseases initiated by S. sclerotiorum ascospores, using tobacco (Nicotiana tabacum) leaf discs as an experimental system (Garriz et al., 2010). The 8^th^-ranking confirmed pair is pathogen Pseudomonas aeruginosa and host Oryctolagus cuniculus. Researchers have determined the pharmacokinetics and adverse effects following SC administration of ceftiofur crystalline free acid (CCFA) in Oryctolagus cuniculus by using Pseudomonas aeruginosa and other bacterium (Gardhouse et al., 2017). The 9^th^-ranking confirmed pair is pathogen Enterococcus faecalis and host Homo sapiens. A study showed that an 88-kDa secreted protein, endoglycosidase (Endo) E, which is most likely responsible for the activity of the human pathogen Enterococcus faecalis, degrades the N-linked glycans of human RNase B to acquire nutrients (Mattias and Fischetti, 2004). The pair of pathogen Alternaria citri and host Citrus reticulate ranks 10th. Reasearchers found that the phytopathogenic fungus, Alternaria citri (Alternaria alternata pathotype citri), produces a complex of analogous toxins (ACTG-toxin) that selectively damages Dancy tangerine (Citrus reticulata) and other mandarin cultivars (Kohmoto et al., 1979). The pair of pathogen Mycobacterium marinum and host Homo sapiens ranks 12th. Flowers found that a person was infected with Mycobacterium marinum by being bitten by a dolphin and thus associated human mycobacterial infection with an aquatic mammal (Flowers, 1970). The 14th score is the pair of pathogen Mycobacteroides abscessus and host Homo sapiens. Mycobacterium abscessus is one of the common species that causes disseminated infections in patients with cystic fibrosis. It has been reported that NLRP3 inflammasome activation contributed to antimicrobial responses against M. abscessus in human macrophages and that its activation was dependent on dectin-1/Syk signaling (Hye-Mi et al., 2012). The pair of pathogen Alternaria alternata and host Solanum lycopersicum ranks 15th. A study evaluated whether 1-MCP treatment could affect postharvest decay caused by A. alternata, B. cinerea, and Fusarium spp. in Solanum lycopersicum (Hai and Gubler, 2012). The 16^th^-ranking association is the pair of pathogen Enterococcus faecium and host Homo sapiens. A previous study was performed to determine whether resistance genes from an E. faecium isolate of animal origin could be transferred to a human E. faecium isolate in the intestines of human volunteers without any selective antimicrobial pressure (Lester et al., 2006). The 17th pair of pathogen and host is Fusarium oxysporum and Nicotiana tabacum. Jennings et al. found that protein Nep1 from Fusarium oxysporum inducted defense responses in tobacco (Jennings et al., 2001). The 19th potential link is Pectobacterium carotovorum and Arabidopsis thaliana. The study indicated that Arabidopsis thaliana were infected with Pectobacterium carotovorum (Lee et al., 2012). The 20th potential link is pathogen Mycoplasma agalactiae and host Mus musculus. Smith G R. et al. used Mycoplasma agalactiae to infect mice to verify the toxicity of the Mycoplasma agalactiae (Smith, 1967). Based on the above findings, one can argue that BNMP is very efficient in predicting associations between pathogens and hosts.

**Table 2:** Pathogen–host pairs predicted using BNMP and their rank according to five methods.

Pathogen	Host	BNMP	NTSMDA	BNP	Zhang's method	WBSMDA
Serratia marcescens	Mus musculus (Iwaya et al., 2005)	1	43	15	17	13
Cronobacter turicensis	Mus musculus (Tóthová et al., 2011)	3	10	26	24	109
Escherichia coli O157:H7	Mus musculus (Tanji et al., 2005)	4	38	172	14	10
Acinetobacter nosocomialis	Homo sapiens (Visca et al., 2011)	5	13	251	119	18
Stenotrophomonas maltophilia	Mus musculus (Zgair and Chhibber, 2011)	6	44	124	21	13082
Sclerotinia sclerotiorum	Nicotiana tabacum (Garriz et al., 2010)	7	61	44	540	169
Pseudomonas aeruginosa	Oryctolagus cuniculus (Gardhouse et al., 2017)	8	588	62	960	55
Enterococcus faecalis	Homo sapiens (Mattias and Fischetti, 2004)	9	37	33	109	19
Alternaria citri	Citrus reticulata (Kohmoto et al., 1979)	10	528	57	9021	41
Mycobacterium marinum	Homo sapiens (Flowers, 1970)	12	39	36	115	26
Mycobacteroides abscessus	Homo sapiens (Hye-Mi et al., 2012)	14	20	25	102	20
Alternaria alternata	Solanum lycopersicum (Hai and Gubler, 2012)	15	261	40	447	3045
Enterococcus faecium	Homo sapiens (Lester et al., 2006)	16	40	27	106	121
Fusarium oxysporum	Nicotiana tabacum (Jennings et al., 2001)	17	118	43	537	1313
Pectobacterium carotovorum	Arabidopsis thaliana (Lee et al., 2012)	19	259	74	199	764
Mycoplasma agalactiae	Mus musculus (Smith, 1967)	20	26	201	101	211

## Discussion

In this study, we focus on the problem of pathogen–host association prediction. To consider the relations of pathogens and hosts comprehensively, we adopt the pattern of local before global, proposing a novel approach, BNMP. The method is based on bipartite network modules and integrates module information of pathogens and hosts, respectively, into a bipartite network projection model to improve prediction performance. Where the host is the seed, the time complexity of acquiring the association score vector between the seed and all pathogens is *O*(*ms*^3^*t*), where *m* is the degree of the seed. Hence, the time complexity of acquiring *S*_*pathogen*−*host*_ is *O*(*es*^3^*t*), where *e* is the number of associations in the host-pathogen association network. Similarly, the time complexity of acquiring $S_{host-pathogen}^{T}$ is *O*(*et*^3^*s*). BNMP has a time complexity of *O*(*est*(*s*^2^+*t*^2^)), namely *O*(*es*^3^*t*) when *s*>*t* and *O*(*et*^3^*s*) when *t*>*s*. Experimental results show that BNMP achieved better prediction performance compared with other efficient methods.

Although BNMP is used here in pathogen–host association prediction, it can also be applied to association analysis in other fields, such as miRNA–disease association prediction, drug–target interaction prediction, and drug–cell line response prediction. Hence, our study has a wide range of uses. Module-based information can help improve the score in the bipartite network because more information related to the nodes in a network is included in the predictive model, which avoid missing the information of neighbors. Although BNMP performs well on the existing data set, the number of associations between pathogens and hosts in the data set is insufficient, which affects the performance of the proposed method. As more association relationships are found or added into databases and more information about regulatory modules (Chen et al., 2019a; Chen et al., 2019b) is employed in the future, the prediction performance of BNMP should further improve.

## Data Availability Statement

Publicly available datasets were analyzed in this study. This data can be downloaded from PHI-base (http://www.PHI-base.org/index.jsp), HPIDB (https://hpidb.igbb.msstate.edu/index.html) and intact (https://www.ebi.ac.uk/intact/).

## Author Contributions

JL and SW designed and implemented the algorithm. ZC and SW analyzed the results and wrote the manuscript, and YW made suggestions. All of the authors read and approved the final manuscript.

## Funding

This work was partially supported by the Natural Science Foundation of Heilongjiang Province (Grant No. F2016016) and the National Key Research and Development Program of China (Grant No.2016YFC0901905).

## Conflict of Interest

The authors declare that the research was conducted in the absence of any commercial or financial relationships that could be construed as a potential conflict of interest.
